# Quantitative evaluation of the beneficial effects in the *mdx* mouse of epigallocatechin gallate, an antioxidant polyphenol from green tea

**DOI:** 10.1007/s00418-012-0926-3

**Published:** 2012-02-14

**Authors:** Yoshiko Nakae, Olivier M. Dorchies, Peter J. Stoward, Benno F. Zimmermann, Christina Ritter, Urs T. Ruegg

**Affiliations:** 1 Pharmacology, Geneva-Lausanne School of Pharmaceutical Sciences, University of Geneva, 30 Quai Ernest Ansermet, 1211 Geneva 4, Switzerland; 2 Institute of Nutrition and Food Sciences, University of Bonn, Endenicher Allee 11-13, 53115 Bonn, Germany; 3 Institut Prof. Dr. Georg Kurz GmbH, Eupener Str. 161, 50933 Cologne, Germany; 4 Present Address: 391 Kamigoryo Banba-cho, Kamikyo-ku, Kyoto, 602-0891 Japan

**Keywords:** Creatine kinase, Epigallocatechin gallate, Fibrosis, Locomotor activity, *Mdx* mouse, Oxidative stress

## Abstract

In two separate previous studies, we reported that subcutaneous (sc) or oral administration of (−)-epigallocatechin-3-gallate (EGCG) limited the development of muscle degeneration of *mdx* mice, a mild phenotype model for Duchenne muscular dystrophy (DMD). However, it was not possible to conclude which was the more efficient route of EGCG administration because different strains of *mdx* mice, periods of treatment and methods of assessment were used. In this study, we investigated which administration routes and dosages of EGCG are the most effective for limiting the onset of dystrophic lesions in the same strain of *mdx* mice and applying the same methods of assessment. Three-week-old *mdx* mice were injected sc for 5 weeks with either saline or a daily average of 3 or 6 mg/kg EGCG. For comparison, age-matched *mdx* mice were fed for 5 weeks with either a diet containing 0.1% EGCG or a control diet. The effects of EGCG were assessed quantitatively by determining the activities of serum muscle-derived creatine kinase, isometric contractions of triceps surae muscles, integrated spontaneous locomotor activities, and oxidative stress and fibrosis in selected muscles. Oral administration of 180 mg/kg/day EGCG in the diet was found the most effective for significantly improving several parameters associated with muscular dystrophy. However, the improvements were slightly less than those observed previously for sc injection started immediately after birth. The efficacy of EGCG for limiting the development of dystrophic muscle lesions in mice suggests that EGCG may be of benefit for DMD patients.

## Introduction

Duchenne muscular dystrophy (DMD) is an X-linked progressive muscle-wasting disease. Its primary defect is mutations and deletions in the dystrophin gene, resulting in a lack of dystrophin at the inner face of the sarcolemma. In normal cells, dystrophin connects the cytoskeletal F-actins to the extracellular matrix through a dystrophin-associated glycoprotein complex in the sarcolemma (Davies and Nowak 2006) and stabilizes the sarcolemma during muscle contractions (Petrof et al. 1993). The absence of dystrophin causes not only mechanical damage in the sarcolemma but also abnormal regulation of reactive oxygen species (ROS), cell signaling and ion channels, followed by elevated calcium influx into myofibres and calcium-dependent proteolysis of myofibrils, and other dysfunctions such as inflammation and apoptosis (Allen et al. 2010; Evans et al. 2009a, b; Tidball and Wehling-Henricks 2007; Whitehead et al. 2006, 2010). This leads to repeated cycles of muscle degeneration and regeneration, and eventually muscle wasting and fibrosis. Currently, the molecular mechanism underlying the pathogenesis of DMD is not fully elucidated and there is no established therapy available.

Increased oxidative stress is a major contributing factor in the pathology of DMD and in dystrophin-deficient muscles in an animal model of DMD, the *mdx* mouse (Rando 2002; Tidball and Wehling-Henricks 2007; Whitehead et al. 2006). For the past decade, several groups, including ours, have investigated the antioxidant effects of a green tea polyphenol mixture (GTP) and its major active constituent (−)-epigallocatechin-3-gallate (EGCG), in *mdx* mice. The main findings reported to date are as follows:

1. *Mdx* mice fed with a diet containing 0.01 or 0.05% GTP for 4 weeks from birth (through their dams before weaning) diminishes necrosis in their extensor digitorum longus (EDL) muscles (Buetler et al. 2002).
2. Feeding 3-week-old *mdx* mice with a diet containing 0.05 or 0.25% GTP or 0.1% EGCG for 1 or 5 weeks increases their antioxidant potential in plasma, reduces necrosis in their EDL muscles, and improves isometric contraction parameters of their triceps surae muscles (Dorchies et al. 2006).
3. Subcutaneous (sc) injection of 5 mg**/**kg EGCG four times a week from birth for 8 weeks limits the onset of muscular dystrophy in *mdx* mice by protecting the sarcolemma from damage as judged by their near normal levels of muscle-derived creatine kinase (CK) in sera, a reduction in the amount of intra- and intermyofibre lipofuscin (LF) or ceroid (Porta 2002), less fibrosis and fewer necrotic myofibres, more histologically normal myofibres, and enhanced expression of utrophin, a homologue of dystrophin (Nakae et al. 2008).
4. Diet containing 0.5% GTP given to *mdx* mice from gestation to 6 weeks after birth increases their voluntary wheel running activities, citrate synthase activities in gastrocnemius muscle, lowers lipid peroxidation in cardiac and gastrocnemius muscles, and decreases sarcolemmal damage (Call et al. 2008). This last EGCG treatment also improves the histopathology of *mdx* tibialis anterior muscles and down-regulates activated NF-κB in the nuclei of the regenerating myofibres (Evans et al. 2010).

The beneficial effects of GTP, and more especially EGCG, on dystrophin-deficient muscles seem to be related to their high antioxidant activity (Higdon and Frei 2003), low molecular weight (458 Da for EGCG), and low toxicity (Chan et al. 2010; Goodin and Rosengren 2003; Isbrucker et al. 2006a, b; McCormick et al. 1999). A major source of ROS in dystrophic *mdx* muscles has been attributed to NADPH oxidase (NOX), expression of which is enhanced in dystrophic muscles (Shkryl et al. 2009; Whitehead et al. 2010). The ROS produced by this enzyme regulate many fundamental physiological events in the cell (Brown and Griendling 2009), but excessive ROS production causes cellular dysfunctions, such as an increase in stretch-induced Ca^2+^ influx into *mdx* myofibres resulting in force reduction (Whitehead et al. 2010). EGCG, epicatechin, and its methylated metabolite also inhibit ROS production by NOX in non-muscle cells (Morré et al*.* 2000; Nishikawa et al. 2007; Steffen et al. 2007, 2008). Moreover, EGCG interacts with plasma membrane 67-kDa laminin receptor (67LR; Tachibana et al. 2004), which is up-regulated in *mdx* muscle cells (Dorchies et al. 2009).

One aim of the present study was to investigate whether sc EGCG administration to *mdx* mice starting 3 weeks after birth, when muscle degeneration had already begun (Coulton et al. 1988; Dangain and Vrbova 1984; Louboutin et al. 1993; Passaquin et al. 2002), was as effective for limiting the onset of dystrophic lesions as EGCG administration begun the day after birth. In addition, the efficacy of different routes and doses of EGCG administration were investigated quantitatively using several assessment criteria. One criterion we used was accumulated oxidative stress in selective muscles, which was determined as the amount of intramuscular LF granules that are formed. LF is an autofluorescent end-product of lipid peroxidation and is widely accepted as an index of chronic oxidative stress (Brunk and Terman 2002; Sohal and Brunk 1989; Terman and Brunk 1998).

We found that EGCG administered either sc or orally to *mdx* mice beginning at 3 weeks after birth leads to similar improvements to those observed previously for sc injection started immediately after birth (Nakae et al. 2008). Of the treatment protocols investigated, oral administration of 180 mg/kg EGCG daily in the diet for 5 weeks was found to be the most effective for reducing muscular dystrophy.

## Materials and methods

### Animals

Dystrophic C57BL/10-*mdx* (*mdx*) mice obtained from Charles River France (Iffa Credo, Lyon) and wild-type (WT) C57BL/10 mice from Charles River Deutschland (Sulzfeld, Germany) and the Jackson Laboratory (Bar Harbor, ME, USA) were used. They were fed with standard laboratory rodent diet pellets, given free access to water, and housed in a room with 12 h light/dark cycles at 20 ± 1°C. Males and females of the same strain were mated and the pregnant females housed in cages lined with soft bedding material (small cotton pads and fine wood chips). For the EGCG experiments, only male littermate neonates were used. They were divided into test and control groups at 3-weeks-old. All experiments were carried out according to the guidelines based on the Swiss Federal Law on Animal Welfare of the Swiss Federal Veterinary Office and were approved by the Cantonal Veterinary Service.

### EGCG administration

(−)-Epigallocatechin-3-gallate (Sunphenon brand EGCG, lot 503310; purity >90%, caffeine <1%), extracted from green tea, was kindly donated by Taiyo Kagaku (Yokkaichi, Japan). For sc administration, it was dissolved in sterile physiological saline (0.9% NaCl) at a concentration of 0.3 or 0.6%. The EGCG solutions were filtered through a sterile syringe 0.20-µm pore filter (DISMIC-13cp; Advantec Toyo, Tokyo, Japan), stored at 4°C, and used within 10 days after preparation. Calculated volumes of the EGCG solution corresponding to a dose of 5 or 10 mg EGCG/kg body weight (bw) were injected sc using autoclaved microsyringes (Hamilton, Reno, Nevada, USA) into the backs of *mdx* mice four times a week for 5 weeks beginning when they were 3-weeks-old. The lower and higher doses of the sc administrations are equivalent to average daily dosages of 3 and 6 mg/kg, respectively (Tables 1, 2). As controls, *mdx* and WT mice were injected with physiological saline only. At the end of a treatment schedule, all assessments, except for locomotor activity, were carried out 24–48 h after the final sc injection as we found during the course of this study that handling of *mdx* mice for injection increases their serum creatine kinase activities. The activities do not reach constant low base levels until at least 24 h after the injection.

**Table 1:** Mean ± SEM of parameters of 8-week-old *mdx* mice and age-matched wild-type (WT) controls after administration of EGCG or saline for 5 weeks by two routes

Mouse:	*Mdx* mice					WT controls	
Administration route:	Subcutaneous			Oral		Subcutaneous	Oral
EGCG dose:	Saline	5 mg/kg	10 mg/kg	Control diet	0.1% EGCG diet	Saline	Control diet
Average daily EGCG-intake per body weight:	0 mg/kg	3 mg/kg	6 mg/kg	0 mg/kg	180 mg/kg	0 mg/kg	0 mg/kg
Serum CK activity × 10^−3^ (U/l)	9.08 ± 1.89 (7)	3.93 ± 0.62 (7)	5.43 ± 1.27 (6)	5.60 ± 0.61 (6)^a^	3.10 ± 0.46 (9)^a^	0.535 ± 0.178 (7)	1.00 ± 0.23 (6)^a^
Force parameters of triceps surae muscle							
*P*_t_ (mN/mm^2^)	59.9 ± 5.0 (9)	76.7 ± 2.8 (9)	74.0 ± 2.7 (9)	65.4 ± 3.7 (8)	86.5 ± 2.6 (10)	121 ± 4 (8)	118 ± 4 (7)
*P*_o_ (mN/mm^2^)	222 ± 17 (9)	287 ± 11 (9)	265 ± 11 (9)	234 ± 9 (8)	320 ± 16 (10)	455 ± 7 (8)	446 ± 19 (7)
Time to peak (ms)	13.8 ± 0.6 (9)	13.6 ± 0.3 (9)	13.3 ± 0.4 (9)	13.8 ± 0.4 (8)	14.2 ± 0.2 (10)	21.0 ± 0.8 (8)	19.4 ± 1.1 (7)
Half relaxation time (ms)	15.4 ± 0.8 (9)	14.2 ± 0.3 (9)	15.0 ± 0.5 (9)	15.6 ± 0.4 (8)	14.2 ± 0.3 (10)	21.0 ± 1.1 (8)	18.4 ± 1.0 (7)
CSA (mm^2^)	11.7 ± 0.6 (9)	11.4 ± 0.3 (9)	11.4 ± 0.3 (9)	12.5 ± 0.3 (8)	11.0 ± 0.3 (10)	10.5 ± 0.5 (8)	10.7 ± 0.4 (7)
Relative locomotor activity	1.00 (9)	1.36 ± 0.16 (8)	1.55 ± 0.18 (6)	1.00 (8)	1.13 ± 0.08 (10)	1.58 ± 0.25 (8)	1.46 ± 0.16 (7)
Number of lipofuscin granules × 10^−4^/mm^3^							
Diaphragm muscle	5.67 ± 0.46 (7)	5.63 ± 0.51 (9)	4.40 ± 0.24 (8)	6.45 ± 0.60 (6)	4.60 ± 0.20 (10)	0.130 ± 0.010 (8)	0.160 ± 0.029 (7)
EDL muscle	0.840 ± 0.170 (8)	0.963 ± 0.101 (9)	1.15 ± 0.10 (9)	0.877 ± 0.064 (8)	1.01 ± 0.07 (10)	0.166 ± 0.016 (8)	0.164 ± 0.028 (7)
Relative area of fibrosis (%) in transverse section of:							
Diaphragm muscle	31.1 ± 1.1 (9)	31.6 ± 0.7 (9)	30.9 ± 1.1 (9)	30.3 ± 0.5 (8)	30.1 ± 0.7 (10)	22.7 ± 0.7 (8)	22.8 ± 0.6 (7)
EDL muscle	22.5 ± 1.5 (8)	21.2 ± 0.6 (9)	20.7 ± 1.6 (9)	19.8 ± 0.8 (8)	17.4 ± 0.5 (10)	15.1 ± 0.8 (8)	14.8 ± 0.8 (7)

*P*_t_ specific phasic twitch tension, *P*_o_ specific maximum tetanic tension, *CSA* cross-sectional area

^a^Mean CK activities at 7-weeks-old

Numbers of mice measured are in parentheses

**Table 2:** Mean differences (%) ± SEM and significances of the mean values of the muscle parameters reported in Table 1

Mouse:	*Mdx* mice			Wild-type controls	
Administration route:	Subcutaneous		Oral	Subcutaneous	Oral
Average daily EGCG-intake:	3 mg/kg	6 mg/kg	180 mg/kg	0 mg/kg	0 mg/kg
	Difference compared to:				
	Saline control	Saline control	Diet control	*Mdx* saline	*Mdx* diet
Serum CK activity	−57 ± 7* (61)	−40 ± 14	−45 ± 8** (55)	−94 ± 2***	−82 ± 4***
Force parameters of triceps surae muscle					
Specific phasic twitch tension (*P*_t_)	+28 ± 5* (27)	+24 ± 4	+32 ± 4*** (40)	+102 ± 7***	+80 ± 6***
Specific maximum tetanic tension (*P*_o_)	+29 ± 5** (28)	+19 ± 5	+37 ± 7** (41)	+105 ± 3***	+91 ± 8***
Time to peak	−1.4 ± 2.0	−3.6 ± 3.2	+2.9 ± 1.3	+52 ± 5***	+41 ± 8***
Half relaxation time	−7.8 ± 2.2	−2.6 ± 3.0	−9.0 ± 1.9	+36 ± 7***	+18 ± 7*
Cross-sectional area (CSA)	−2.6 ± 2.6	−2.6 ± 2.2	−12 ± 2* (86)	−10 ± 4	−14 ± 4**
Spontaneous locomotor activity	+36 ± 16* (62)	+55 ± 18* (95)	+13 ± 8	+58 ± 25*	+46 ± 16*
Number of lipofuscin granules					
Diaphragm muscle	−0.7 ± 8.9	−22 ± 4	−29 ± 3*** (30)	−98 ± 1***	−98 ± 1***
EDL muscle	+15 ± 12	+37 ± 12	+16 ± 8	−80 ± 2**	−81 ± 3***
Fibrosis					
Diaphragm muscle	+1.6 ± 2.3	−0.6 ± 3.7	−0.7 ± 2.1	− 27 ± 2***	−25 ± 2***
EDL muscle	−5.8 ± 2.7	−8.0 ± 7.1	−12 ± 2* (48)	−33 ± 4**	−25 ± 4***

The recovery scores (%) calculated for the significant differences are in parentheses

Significances: *** P < 0.001, ** 0.001 ≤ *P* ≤ 0.01, * 0.01 < *P* ≤ 0.05. Where no significance is indicated, the difference was not significant

For oral administration of EGCG, *mdx* mice were fed for 5 weeks, beginning when they were 3-weeks-old, with standard rodent diet pellets containing 0.1% EGCG prepared by Provimi Kliba (Kaiseraugst, Switzerland). *Mdx* and WT mice fed with standard diet were used as oral administration controls. Body weights and chow consumption of *mdx* mice were measured weekly. The daily average diet consumption (g) per gram bw was calculated from the food consumed at the end of each week.

### Serum creatine kinase (CK) assays

Blood sampling for CK assays was carried out by saphenous vein puncture (Hem et al*.* 1998). The left lateral ankle was shaved and the lateral saphenous vein pricked with a sterile needle (25 G × 16 mm). The drop of blood (about 20 μl) was collected with a pipette fitted with a sterile tip. The procedure was completed within 3**–**4 min after first handling a mouse. The blood samples were kept at room temperature for 30 min and then centrifuged at 1,000×*g* at 4°C for 20 min to obtain sera. The sera were stored at 4°C and used within 3 days for CK assays. The serum CK activities were measured as the initial velocities of NADPH formation at 37°C over 10 min in a microplate reader (Fluostar Optima; BMG Labtech Sarl, Champigny-sur-Marne, France) using a commercial CK assay kit (Catachem, Bridgeport, CT, USA) according to the manufacturer’s instructions. Catatrol I (Catachem) was used as a positive control. The activities were expressed as U/l.

### Spontaneous locomotor activity

At the end of a treatment schedule, spontaneous locomotor activities of test and control mice were measured simultaneously in separate transparent-plastic cages using a LOCOMO sensor system consisting of LOCOMO sensor units (LS-5), a counter interface (CIF-mini4), a control unit (LCU-20) and a personal computer with WinCIF II mini software (Melquest, Toyama, Japan). The counter interface counted the interruptions of latticed infrared-beams by a mouse moving in the cage inside the sensor. The locomotor activity was expressed as the counts integrated for 12 h from 19.00 h to 7.00 h in the dark phase. The relative locomotor activity of a test *mdx* mouse was expressed as the ratio of its activity to that of a control mouse measured simultaneously to minimize the effects of circumstances and condition of the mouse on the activity.

### Isometric force measurements

Animals were anaesthetized by intraperitoneal injection of a mixture of urethane (1.5 g/kg bw) and diazepam (5 mg/kg bw) in saline. The Achilles tendon of the right hind limb was exposed and linked to a force transducer coupled to a LabView interface for trace acquisition and analysis. The knee joint was firmly immobilized. Two thin steel electrodes were inserted into the triceps surae muscle. The muscles were electrically stimulated with 0.5-ms square pulses of controlled intensity and frequency. The stimulation-recording protocol and data analyses were performed as described previously (Dorchies et al. 2006; Reutenauer et al. 2008; Hibaoui et al. 2011). Absolute phasic and tetanic tensions were converted into specific tensions (mN per mm^2^ of muscle section) after normalization for the muscle cross-sectional area (CSA). The CSA values (mm^2^) were determined by dividing the triceps surae muscle mass in mg, by the product of the optimal length *L*_o_ in mm and *d*, the density of mammalian skeletal muscle (1.06 mg/mm^3^).

### Preparation of tissue sections

After completion of the force measurements, blood was collected from the heart (see below). The left costal part of the diaphragm muscle, the left extensor digitorum longus (EDL) muscle, liver and right kidney were excised, immediately placed in PolyFreeze (Polysciences, Warrington, PA, USA) and quenched in isopentane cooled by liquid nitrogen. Transverse sections, 7-μm-thick, were cut at −20°C at the mid-belly of the EDL muscles and in the middle region (corresponding to mid-belly) of the diaphragm muscles in a cryostat (Cryo-Star HM 560 M; MICROM International, Walldorf, Germany), dried at room temperature and kept at −80°C until used for histology or histochemistry. Seven-μm-thick sections of liver and kidney were prepared similarly.

### Oxidative stress

The levels of accumulated oxidative stress in individual muscles were estimated by counting the numbers of autofluorescent LF granules they contained as described previously (Nakae et al. 2001, 2004, 2008) in sections of diaphragm and EDL muscles mounted in a mixture of 13.3% Mowiol 4-88 Reagent (CalBiochem, San Diego, CA, USA) and 33.3% glycerol (Fluka, Buchs, Switzerland) in 0.133 M Tris–HCl buffer, pH 8.5. In preliminary experiments, we found that the relative sectional area occupied by the granules, whose Feret diameters were mostly between 2 and 5 μm, correlated strongly with their counts per unit area, thus validating this methodology.

One muscle section prepared as described in the previous paragraph was used for the measurement in each muscle. Emission signals at 515 nm from the section excited at 450–490 nm were captured as 50–60 or 10–15 images for one whole section of diaphragm or EDL muscle, respectively, using a Spot Insight B/W camera (model 3.1, Visitron Systems, Puchheim, Germany), fitted to a fluorescence light microscope with a ×20 objective (Zeiss Axiovert 200 M; Carl Zeiss MicroImaging, Jena, Germany). The total number of LF granules and tissue area were determined in every captured image using the ‘cell counter’ function and ‘measure area’ function, respectively, of ImageJ free software version 1.41o (NIH, Maryland, USA). Assuming that the muscle sections were uniformly 7-μm thick, the number of LF granules per mm^3^ of diaphragm or EDL muscle was calculated for each mouse. Microphotographs of serial sections stained with Mayer’s haematoxylin and eosin were taken with a Spot Insight QE camera (model 4.2; Visitron Systems) fitted to the same light microscope. MetaView software (Visitron Systems) was used for capturing both colour and black and white images.

### Fibrosis

To evaluate the fibrosis in each muscle, the amount of intermyofibre connective tissue formed in selected muscles was quantified in situ by utilizing the specific binding of wheat-germ agglutinin (WGA) to *N*-acetylglucosamine and sialic acid residues in proteoglycans, polysaccharides and glycoproteins in the extracellular matrix (Dunn et al. 1982; Kostrominova 2011). Transverse sections of diaphragm and EDL muscles, prepared as described previously, were labelled with 2 μg/ml WGA-Alexa Fluor 594 conjugate (Molecular Probes, Eugene, OR, USA) in phosphate-buffered saline (PBS), pH 7.4, at room temperature for 1 h according to a modification of the method described by Briguet et al. (2004). The labelled sections were rinsed for 10 min in two changes of ice-cold PBS and then mounted in the Mowiol mounting medium referred to earlier. Emission signals at 615 nm from the labelled sections excited at 530–585 nm were captured as 10–23 or 3–6 images for respectively one whole section of the left costal part of diaphragm or left EDL muscle for each mouse using a ×10 objective as described in the previous paragraph. Using the ImageJ version 1.41o software, the percentage of WGA-labelled tissue area of the total tissue area in each section of diaphragm or EDL muscle was determined in the captured images converted to binary images by applying the automatic threshold function of the software. However, the connective tissue in the epimysium was excluded from the quantification as only the fibrotic deposits in the endomysium and perimysium are relevant for DMD pathogenesis. Serial sections were stained with Mayer’s haematoxylin and eosin.

### Plasma EGCG assays

Immediately after the force measurements of the triceps surae muscle had been completed, the chest cavity was exposed. 10 μl 5% heparin sodium salt (147 USP units/mg; Sigma, St. Louis, MO, USA) in saline was injected into the heart, and a blood sample for HPLC analysis taken by cardiac puncture. The blood samples were kept at room temperature for 30 min and then centrifuged at 1,000×*g* at 4°C for 20 min to obtain the plasma. The plasma samples were stabilised by adding 10 μl antioxidant mixture per 100 mg plasma. The antioxidant solution consisted of 20% ascorbic acid and 0.1% EDTA in 0.4 M NaH_2_PO_4_–H_3_PO_4_ buffer, pH 3.6 (Lee et al. 2000). The samples were stored at −80°C until use.

The EGCG concentrations in the plasma samples were determined according to the method of Zimmermann et al. (2009). The samples were thawed and their sulphated and glucuronidated EGCG content deconjugated by adding to 200 μl aliquots, 50 μl of an enzyme suspension containing 40 U sulphatase (type VIII, EC 232-772-1, purified from abalone entrails; Sigma-Aldrich, Steinheim, Germany) and 500 U β-glucuronidase (type VII-A, EC 232-606-8, purified from *E. coli*, Sigma-Aldrich) followed by incubation at 37°C for 45 min. Proteins were precipitated by adding 100 μl dimethylformamide and 20 μl 50% (w/w) aqueous trichloroacetic acid to the treated plasma, and then separated by centrifugation at 8,800×*g* for 7 min. Twenty μl of the supernatant was injected into a HPLC. The total EGCG content was identified and quantified by HPLC with electrochemical detection as described in Zimmermann et al. (2009)*.* Unconjugated (free) EGCG was determined similarly in parallel samples to which no enzyme suspension had been added. The lowest limit of EGCG quantification was 5.0 ng/ml plasma, recovery 96.5%, and the coefficient of variation 8.6%.

### Data analyses and recovery scores

**Fig. 1:**
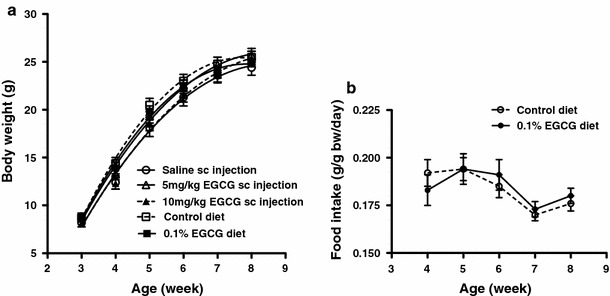
Mean body weights (**a**) and daily average food intake per body weights (**b**) of *mdx* mice during EGCG administration from age 3 to 8 weeks. **a** When compared with the corresponding control group, there was no significant difference in the mean body weights of the sc injection groups (*n* = 9 for each group) and oral administration groups (*n* = 8 or 10) throughout the treatment. **b** 0.1% EGCG in the diet has no significant effect on food intake by *mdx* mice (*n* = 8 for control group and *n* = 10 for test group). The daily EGCG delivery by the diet route averaged 180 mg/kg bw

Data were expressed as means ± SEM and analysed using GraphPad Prism software version 5.0c (GraphPad Software, La Jolla, CA, USA). A two-tailed paired Student’s *t* test (for data on the relative locomotor activities in *mdx* mice in Fig. 4b) or a two-tailed unpaired Student’s *t* test (for comparison in oral administration groups in Fig. 1 and comparison between *mdx* controls either with or without saline injection and corresponding WT controls in Fig. 4a) was used to assess statistical significances between two means. Whenever more than three groups of *mdx* and WT mice treated by the same administration route had to be compared, their data were subjected to one-way ANOVA followed by Tukey’s multiple-comparison test to compare all pairs of the groups. Differences were considered significant at *P* ≤ 0.05. Significance levels are denoted as follows: ***P < 0.001; **0.001 ≤ *P* ≤ 0.01; *0.01 < *P* ≤ 0.05.

The mean differences ± SEM in the parameters between test *mdx* mice or WT controls and corresponding *mdx* controls treated by the same route were denoted by *D*_t_ and *D*_o_, respectively, and expressed as percentage (Table 2). Statistically significant improvements of the parameters after EGCG treatment were expressed as recovery scores (%) calculated as (*D*_t_/*D*_o_) × 100 (Table 2) as proposed by Gillis (2002).

The correlation of locomotor activity with LF formation or fibrosis in muscles was analysed using the same Prism software.

## Results

### Effects of the EGCG treatments on mouse body weights and food intake

Neither sc nor oral EGCG treatments for 5 weeks had any significant effects on the mean body weights of *mdx* mice as compared to the corresponding controls (Fig. 1a). Similarly, the mean daily food intake was unaffected by the treatments (Fig. 1b). It was in the range 0.170–0.194 g/g bw/day for the control diet mice and 0.173–0.194 g/g bw/day for mice fed with the 0.1% EGCG diet. The daily average EGCG intake by the dietary route was equivalent to 180 mg/kg bw in *mdx* mice and was 20% higher than that reported previously for *mdx* ^*5Cv*^ mice, another mutant strain (Dorchies et al. 2006).

### Effects of handling and injection procedures on serum creatine kinase (CK) levels

**Fig. 2:**
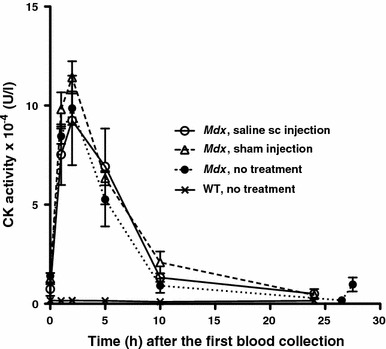
Time courses of the mean serum CK activities in single mice measured after different handling procedures. Just before the first blood sample was drawn at time zero, 9-week-old male *mdx* mice were injected either with 1.67 ml/kg physiological saline (0.9% NaCl) sc or sham injected. Other age-matched male *mdx* mice were not injected (no treatment group). Blood samples were then drawn from the same mice over a period of 24–27.5 h, and their serum CK activities measured (*n* = 4 for each treatment group). The mean CK activities of four age-matched male WT mice not subjected to any procedure were also determined. For all experimental groups of *mdx* mice, the CK activities rose sharply during the first 2 h after first blood drawing from the mice but declined steadily over the following 8 h, reaching pre-stress levels after 24 h

To obtain reliable data on the changes in CK levels in individual mice during EGCG treatment, the effects of handling mice, saline sc injection and the blood drawing procedure on serum CK levels were first examined. This enabled the best time window for blood collection after a sc injection to be determined. Just before the first blood sample was drawn from the lateral saphenous vein at time zero, 9-week-old male *mdx* mice were divided into three groups. The first group was injected with physiological saline (1.67 ml/kg) sc at the same dose used in the EGCG treatment protocol. The second group was sham-injected and the third group received no prior treatment. The time-course of serum CK activity was measured in each mouse over a 24**–**27.5 h period after the first blood collection at time zero. The mean CK activities of the three *mdx* groups in blood samples withdrawn immediately, or shortly after, a sc injection ranged from 7,400 to 14,000 U/l. They increased 7- to 12-fold in samples withdrawn over the next 2 h, but then decreased exponentially and returned to the original levels after 24 h (Fig. 2). Similar time courses of CK activities were observed in the three groups of *mdx* mice subjected to different injection procedures, suggesting that the changes were due to the trauma caused by the first blood withdrawal. In contrast, age-matched male WT mice without pre-treatment showed no such changes in their serum CK activities after the first blood sampling (Fig. 2). Subsequently, all blood samples for CK assays in this study were collected via the left lateral saphenous vein at least 24 h after a previous sc injection or blood sampling.

### Serum CK activities monitored during EGCG administration

**Fig. 3:**
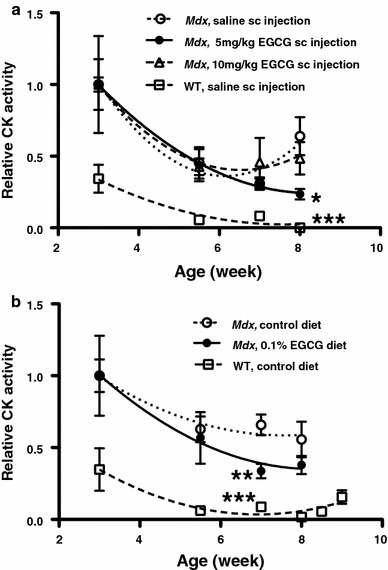
Mean relative CK activities in sera determined continuously in the same *mdx* mice given EGCG **a** sc (*n* = 4–7 for each experimental point) or **b** orally in the diet (*n* = 4–10 for each experimental point). The serum CK levels at each age point of control WT mice treated with saline (*n* = 7 or 8) or a control diet (*n* = 4–6) were normalized by dividing the activities by the mean CK activities in 3-week-old *mdx* mice (11,300 U/l) before EGCG treatment. When compared with the activities in corresponding *mdx* controls, the mean relative CK activities of *mdx* mice were reduced significantly by 57 ± 7% (*n* = 7) after 5-week sc administration with the lower dose of EGCG and by 45 ± 8% (*n* = 9) after 4-week oral administration of EGCG. Significance levels denoted as ***P < 0.001; **0.001 ≤ *P*≤ 0.01; *0.01 < *P* ≤ 0.05 are for the comparison to corresponding *mdx* controls

Before EGCG treatment, the mean serum CK activities in 3-week-old *mdx* mice (11,300 ± 1,050 U/l, *n* = 34) were about threefold higher than that of age-matched WT mice (3,890 ± 880 U/l, *n* = 13). The time courses of the serum CK levels during EGCG treatment are shown in Fig. 3 where the CK levels in *mdx* mice are expressed as the mean activities relative to the mean CK activities of 3-week-old *mdx* mice in each treatment group before EGCG treatment. The CK levels in WT mice with or without saline injection were expressed as the mean activities relative to the mean CK activities of 3-week-old *mdx* mice before EGCG treatment. The mean CK levels in untreated *mdx* and WT mice decreased as they grew older. During the first 3 weeks of EGCG treatment, whether by the sc or oral route, the mean CK levels in *mdx* mice fell similarly to their respective controls. In contrast, the mean CK activity in *mdx* mice given 5 mg/kg EGCG 4 times a week sc for 2 weeks longer was reduced significantly by 57 ± 7% (Fig. 3a; Tables 1, 2). The recovery score was 61% (Table 2). The mean CK level in *mdx* mice given a higher dose (10 mg/kg EGCG 4 times a week) by the same sc injection protocol showed less reduction, 40 ± 14% at the end of sc treatment period, compared to the saline-treated control (Fig. 3a; Tables 1, 2). However, this reduction was not statistically significant. Feeding 3-week-old *mdx* mice with 0.1% EGCG in their diet for 4 weeks reduced their mean serum CK activity highly significantly by 45 ± 8% as compared to that of untreated *mdx* controls (Fig. 3b; Tables 1, 2). The recovery score was 55% (Table 2). In contrast, oral EGCG treatment of *mdx* mice for a week longer resulted in a smaller and non-significant reduction (26 ± 12%) in the mean CK activities (Fig. 3b).

### Effects of EGCG treatments on isometric contraction parameters

Sc injection of 5 mg/kg EGCG for 5 weeks significantly increased the mean specific phasic twitch tension (*P*_t_ = 59.9 ± 5.0 mN/mm^2^) and specific maximum tetanic tension (*P*_o_ = 222 ± 17 mN/mm^2^) of triceps surae muscles of 8-week-old saline-injected *mdx* mice by 28 ± 5 and 29 ± 5%, respectively (Tables 1, 2). The recovery scores for the *P*_t_ and *P*_o_ were 27 and 28%, respectively (Table 2). When compared with *mdx* mice given the control diet, oral EGCG administration also increased *P*_t_ (65.4 ± 3.7 mN/mm^2^) and *P*_o_ (234 ± 9 mN/mm^2^) highly significantly by 32 ± 4 and 37 ± 7%, respectively (Tables 1, 2). The recovery scores for *P*_t_ and *P*_o_ were 40 and 41%, respectively (Table 2). In contrast, sc injection of 10 mg/kg EGCG did not significantly alter either *P*_t_ or *P*_o_ (Tables 1, 2). Other isometric force parameters of *mdx* triceps surae muscles, such as the time to twitch peak and the time for half-relaxation from the peak, were both significantly lower (*P* < 0.001) than those of the corresponding WT muscles, but neither oral or sc EGCG administration had any significant effect on these parameters (Tables 1, 2). The EGCG treatments also did not significantly alter tension–frequency relationships, muscle resistance to repetitive tetanization (an assay for evaluating fatigue) and phasic-to-tetanic ratios in *mdx* triceps surae muscles (data not included here). The mean cross-sectional area of the triceps surae muscles in *mdx* mice was reduced significantly by 12 ± 2% to almost the normal level after oral EGCG treatment (Tables 1, 2) but not by ECGC administered by sc routes.

### Effects of the EGCG treatments on spontaneous locomotor activities

**Fig. 4:**
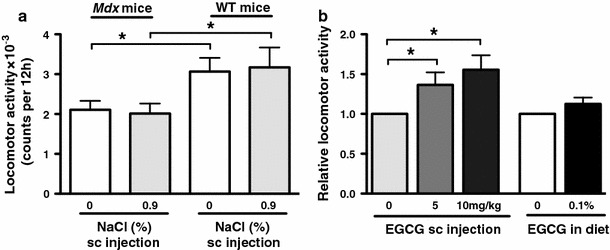
**a** Mean integrated spontaneous locomotor-activities of 8-week-old *mdx* (*n* = 8 or 9) and WT (*n* = 7 or 8) control mice in the dark phase and **b** the effects of EGCG treatments on the relative locomotor activities of *mdx* mice as compared to corresponding controls (*n* = 6–10 per group). Sc injection of 5 and 10 mg/kg EGCG significantly increased the mean relative locomotor activities by 36 ± 16 and 55 ± 18%, respectively. *0.01 < *P* ≤ 0.05

Sc saline injection alone 4 times a week for 5 weeks had no significant effects on the mean integrated locomotor activity of either 8-week-old *mdx* or WT mice (Fig. 4a). The mean locomotor activity (2,060 ± 160 counts per 12 h dark phase, *n* = 17) of *mdx* mice, with or without saline sc injection, was significantly 34 ± 5% lower than that (3,120 ± 300 counts, *n* = 15) of the corresponding WT mice (Fig. 4a). Sc injection of 5 mg/kg and 10 mg/kg EGCG resulted in significant and dose-dependent increases, by 36 ± 16 and 55 ± 18%, respectively, in the relative locomotor activities (compared to that of saline-injected *mdx* mice measured simultaneously) (Fig. 4b; Tables 1, 2). The recovery scores after treatments with the lower and higher doses of EGCG were 62 and 95%, respectively (Table 2). Feeding with 0.1% EGCG increased the locomotor activities of *mdx* mice less, by 13 ± 8%: this increase was not statistically significant (Fig. 4b; Tables 1, 2).

### Effects of EGCG treatments on oxidative stress in muscles

**Fig. 5:**
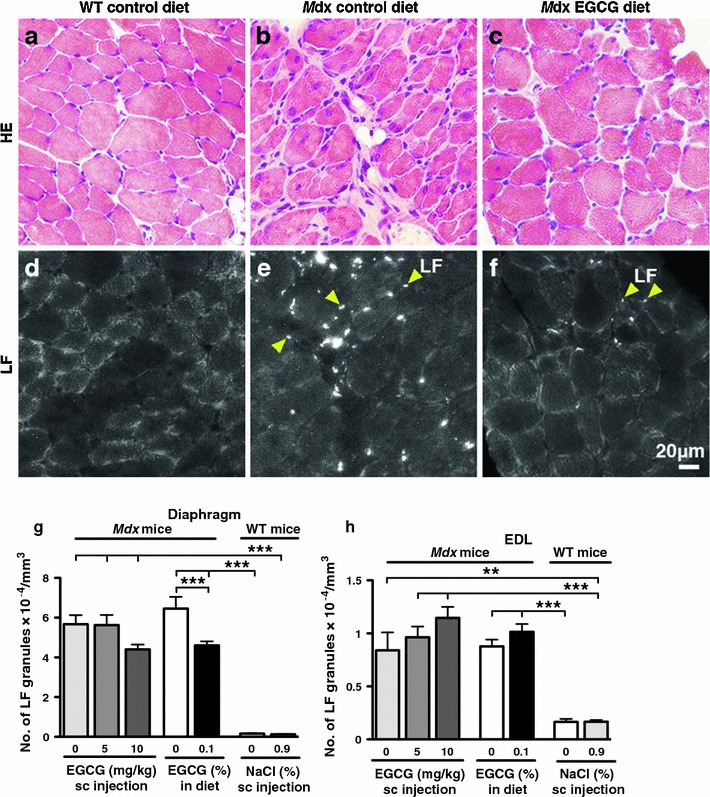
Effects of EGCG on LF formation in diaphragm (**b**, **c**, **e**, **f**, **g**) and EDL (**h**) muscles of 8-week-old *mdx* mice at the end of EGCG treatment schedules. **a**–**c** Diaphragm muscle sections stained with haematoxylin and eosin. **d**–**f** Autofluorescent images of serial muscle sections. *Mdx* control muscles show the typical muscle degeneration and regeneration (**b**) and abundant LF granules (*white dots* in **e**), a product of oxidative stress (**g**, **h**). LF granules are rarely seen in muscles in age-matched WT mice (**a**, **d**, **g**, **h**). In diaphragm muscles of *mdx* mice given 0.1% EGCG in their diet, the histology of the muscles is more normal, and the mean number of LF granules is significantly 29 ± 3% (*n* = 10) less than in *mdx* controls (**c**, **f**, **g**). In contrast, none of the EGCG treatments had a significant effect on LF formation in *mdx* EDL muscles (**h**). **g** *n* = 6–10 per group, **h** *n* = 7–10 per group. ***P < 0.001, **0.001 ≤ *P* ≤ 0.01

Figure 5 shows the effects of EGCG treatments on LF formation in *mdx* diaphragm and EDL muscles. Autofluorescent LF granules were rarely seen in these muscles of 8-week-old WT mice (Fig. 5d, g, h; Table 1; Nakae et al. 2001, 2004). In contrast, as reported previously (Nakae et al. 2001, 2004, 2008), very high numbers of LF granules (60,300 ± 3,710/mm^3^, *n* = 13) were observed focally in myofibres and interstitial cells in the regions with typical features of muscle degeneration and regeneration in diaphragm muscles of age-matched *mdx* controls either with or without saline sc injection (Fig. 5b, e, g; Table 1). Feeding with 0.1% EGCG in the diet for 5 weeks reduced the mean number of LF granules by 29 ± 3% in *mdx* diaphragm muscles (Fig. 5f, g; Tables 1, 2). The reduction was highly significant (*P* < 0.001). The recovery score was 30% (Table 2). In contrast, although sc EGCG administration, particularly at the higher dose tested, reduced LF accumulation by 22 ± 4% in *mdx* diaphragm muscles, the reduction was not significant (Fig. 5 g: Tables 1, 2).

EDL muscles of *mdx* untreated controls and controls saline-injected contained far fewer LF granules (8,580 ± 880/mm^3^, *n* = 16) (Fig. 5 h; Table 1) compared to the corresponding diaphragm muscles. Further, neither oral nor sc administration of EGCG had any significant effect on the mean LF number in EDL muscles though any EGCG treatment appears to increase the number slightly (Fig. 5 h; Tables 1, 2).

The number of LF granules per unit area was found to correlate strongly with the percentage area they occupied, as also reported by Tohma et al. (2011), and thus is a reliable parameter for the amount of LF that is formed: a plot of the areas for LF granules in *mdx* and WT muscles against counts showed a linear relationship with a high correlation coefficient (*R* ^2^ = 0.924; Nakae et al. in preparation).

### Effects of EGCG treatments on fibrosis in muscles

**Fig. 6:**
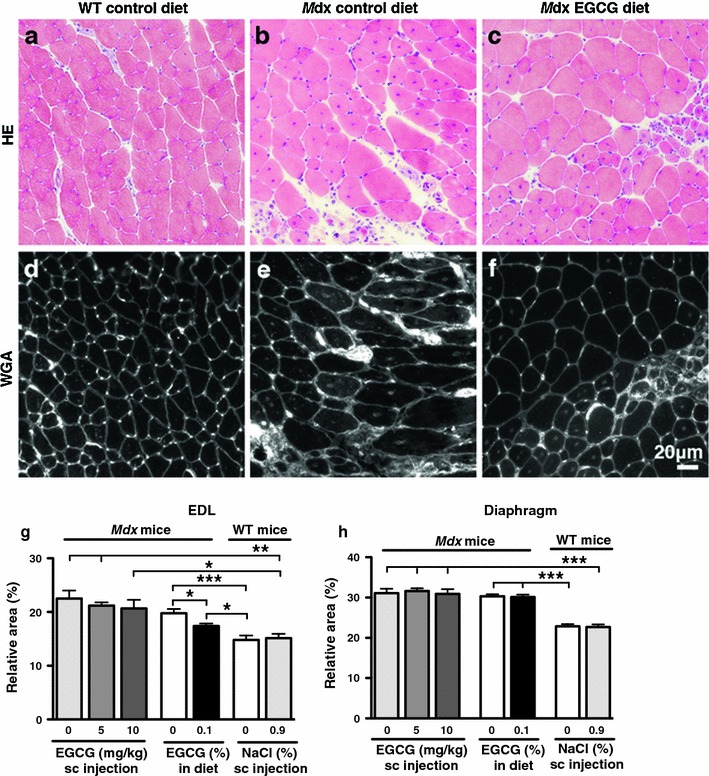
Effects of EGCG on fibrosis (connective tissue formation) in EDL (**b**, **c**, **e**, **f**, **g**) and diaphragm (**h**) muscles of 8-week-old *mdx* mice at the end of EGCG treatment schedules. **a**–**c** EDL muscle sections stained with haematoxylin and eosin. **d**–**f** Connective tissue labelled with WGA-Alexa Fluor 594 conjugate in serial muscle sections. Significantly more connective tissue is seen in the perimysium and endomysium in *mdx* control muscles (**b**, **e**, **g**, **h**) than in muscles in age-matched WT mice (**a**, **d**, **g**, **h**). Administration of 0.1% dietary EGCG significantly diminished the relative area of fibrosis by 12 ± 2% (*n* = 10) in *mdx* EDL muscle (**c**, **f**, **g**), but not in *mdx* diaphragm muscle (**h**). **g** *n* = 7–10 per group, **h** *n* = 7–10 per group. ***P < 0.001, **0.001 ≤ *P* ≤ 0.01, *0.01 < *P* ≤ 0.05

As Fig. 6a–f illustrates, the main structure labelled with WGA-Alexa Fluor 594 conjugate in sections of skeletal muscle was the connective tissue surrounding either the myofibres (consisting of the endomysium and perimysium) or the entire muscle (consisting of the epimysium). Muscle spindles, nerve bundles and blood vessels present in the connective tissue were also labelled as reported previously (Dunn et al. 1982; Kostrominova 2011). In addition to the connective tissue, cytoplasmic clumps in regenerating myofibres and centrally located nuclei of immature and regenerated myofibres were also labelled by the WGA conjugate. The mean ± SEM of the relative areas of labelled cytoplasmic clumps and central nuclei to the total labelled areas accounted for as little as 2.88 ± 0.66% (*n* = 15) and thus was considered negligible.

The mean relative area of connective tissue in sections of EDL muscles was about 30% significantly higher in *mdx* mice than that in WT mice (Fig. 6 g; Tables 1, 2). Oral but not sc EGCG administration significantly reduced the fibrosis of *mdx* EDL muscles by 12 ± 2% (Fig. 6e–g; Tables 1, 2). The recovery score was 48% (Table 2). In sharp contrast, none of the EGCG treatments had a significant effect on the fibrosis of *mdx* diaphragm muscle although the amount of connective tissue in this muscle was about 25% significantly higher compared to the WT muscle (Fig. 6 h; Tables 1, 2).

### Relationship of spontaneous locomotor activity and oxidative stress or fibrosis

**Fig. 7:**
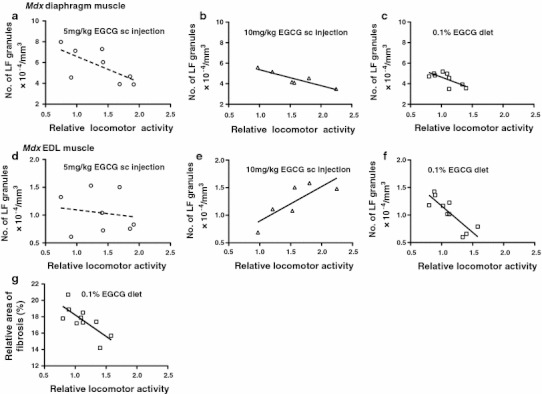
Relationship of spontaneous locomotor activity to LF formation (an index of oxidative stress **a**–**f**) or the relative area of connective tissue (an index of fibrosis **g**) in 8-week-old *mdx* mice at the end of EGCG treatment schedules. **a**–**c** Diaphragm muscles, **d**–**g** EDL muscles. The *solid* and *dashed* *correlation regression lines* show significant (*P* ≤ 0.05) and non-significant correlations, respectively, between the two variables. The relationships are approximated by: **a** *y* = −24,800*x* + 90,600 (*R* ^2^ = 0.455, *P* = 0.0667, *n* = 8), **b** *y* = −15,500*x* + 69,100 (*R* ^2^ = 0.820, *P* = 0.0129, *n* = 6), **c** *y* = −22,300*x* + 68,800 (*R* ^2^ = 0.502, *P* = 0.0327, *n* = 9), **d** *y* = −1,360*x* + 12,300 (*R* ^2^ = 0.0245, *P* = 0.711, *n* = 8), **e** *y* = 6,260*x* + 2,680 (*R* ^2^ = 0.661, *P* = 0.0492, *n* = 6), **f** *y* = −9,620*x* + 21,300 (*R* ^2^ = 0.717, *P* = 0.00200, *n* = 10), and **g** *y* = −5.23*x* + 23.5 (*R* ^2^ = 0.554, *P* = 0.0136, *n* = 10). The negative slopes of the lines in **b**, **c**, and **g** suggest that increases in locomotor activities by EGCG administration correlate significantly with decreases in oxidative stress in diaphragm muscles or fibrosis in EDL muscles. In contrast, the slope of the line for the correlation of locomotor activity with oxidative stress in EDL muscle is positive (**e**) or negative (**f**) depending on the routes and doses of EGCG administration

The mean numbers of LF granules formed in *mdx* diaphragm or EDL muscle (*y* axis) correlated significantly with the corresponding mean relative locomotor activities (*x* axis) in *mdx* mice sc injected with 10 mg/kg EGCG or fed with 0.1% EGCG diet (Fig. 7b, c, e, f). The equations of the correlation regression lines are indicated in the figure legends. In contrast, there was no significant correlation between these variables in *mdx* mice sc injected with 5 mg/kg EGCG (Fig. 7a, d).

The relative areas of intermyofibre connective tissue (fibrosis) in EDL muscles (*y* axis) plotted on the relative locomotor activities (*x* axis) showed a significant correlation only for *mdx* mice given 0.1% dietary EGCG (Fig. 7 g). The same plots for EDL and diaphragm muscles in sc EGCG-injected *mdx* mice and those for diaphragm muscles in *mdx* mice given EGCG sc or orally showed no significant correlations because the changes in fibrosis by the treatments were too small.

### Plasma EGCG concentrations

The mean ± SEM concentrations of free and total EGCG determined in plasma in *mdx* mice fed with 0.1% EGCG diet for 5 weeks were 27.6 ± 3.2 ng/ml (*n* = 10) and 29.7 ± 3.9 ng/ml (*n* = 10), respectively. These values were not significantly different. The mean concentration of conjugated EGCG was 2.08 ± 2.19 ng/ml (*n* = 10). For EGCG administered by sc routes, the EGCG concentrations in *mdx* plasma samples (*n* = 10) obtained 24–48 h after at the end of a treatment schedule were below the limit of quantification by HPLC.

### Effects of EGCG treatments on organ histology

At the light microscopical level, no histological changes were observed in haematoxylin–eosin stained sections of livers (*n* = 10) and kidneys (*n* = 10) of 8-week-old *mdx* mice fed with 0.1% EGCG in their diet for 5 weeks.

## Discussion

In this paper, we report that treatment of *mdx* mice, beginning when they are 3-weeks-old, with EGCG, either injected sc (3 mg or 6 mg/kg/day) or given orally in their diet (180 mg/kg/day), delays or limits the development of dystrophic lesions. The improvements in muscle function and structure are similar to those observed previously for sc injection (3 mg EGCG kg/day) started immediately after birth (Nakae et al. 2008), although there were some differences. The EGCG doses delivered in the present study were not toxic: they had no effect on body weight (Fig. 1a), food intake (Fig. 1b) or liver and kidney histology. The absence of adverse effects agrees with our previous reports (Dorchies et al. 2006; Nakae et al. 2008). In contrast, we found that one-day-old *mdx* pups died within 24 h after a single sc injection of 25 mg/kg EGCG (Nakae et al., unpublished data). Hepatonecrotic responses have been reported when much higher EGCG doses were administered, for example 50 mg EGCG kg/day injected intraperitoneally into adult mice for 7 days (Goodin and Rosengren 2003). Therefore, sc doses of EGCG higher than 10 mg/kg were not used in our investigation to avoid their adverse effects.

We were concerned that repeated handling and injections may affect serum CK levels of *mdx* mice. Consequently, we first determined the time course of CK activities over 24 h after an initial blood withdrawal (Fig. 2). We found that the initial handling of *mdx* mice, but not WT mice, causes substantial increases in the serum CK levels during the first 1**–**2 h. This was followed by a progressive return to the initial levels by 24 h. Therefore, to avoid handling-mediated artefacts, blood samples for CK assay were collected 24 h after a sc injection. Our finding of variable rises in CK levels in *mdx* mice subjected to mild trauma is consistent with previous reports (e.g. Grounds et al. 2008).

By avoiding the stress-induced CK changes, we found that the serum CK levels in untreated *mdx* and WT mice decrease with age from 3 to 8 weeks (Fig. 3b), agreeing with earlier findings (Coulton et al. 1988). The age dependency of CK release mirrors the changes with age of the relative area of degenerating myofibres in *mdx* tibialis anterior muscle (Evans et al*.* 2010), indicating that serum CK activity is a marker that closely parallels sarcolemmal damage followed by necrosis. Oral administration of 180 mg EGCG/kg/day or a sc injection protocol delivering on average 3 mg EGCG/kg/day to young *mdx* mice for 4 or 5 weeks significantly recovered serum CK levels towards normal levels by 55–61% (Table 2). However, this improvement was not as great as the 75% recovery to normal levels observed previously in age-matched *mdx* mice injected sc from birth with an average 3 mg EGCG/kg/day (Nakae et al. 2008). This suggests that the EGCG treatment started prior to the onset of massive necrosis in most *mdx* muscles, which occurs about 3–4 weeks after birth (Coulton et al. 1988; Dangain and Vrbova 1984; Louboutin et al. 1993; Passaquin et al. 2002), protects the sarcolemma of *mdx* myofibres effectively. This is supported by previous evidence that *mdx* muscles are subjected to free-radical mediated injury even during the pre-necrotic state (Disatnik et al. 1998).

Sc injection of the lower dose of EGCG (but not the higher dose) and oral administration of EGCG led to recoveries of the deficit in specific phasic twitch tensions (*P*_t_) of *mdx* triceps surae muscles by 27 and 40%, respectively, compared to normal muscles. Similarly, the deficit in specific tetanic tensions (*P*_o_) recovered by 28 and 41%, respectively (Table 2). Oral administration of EGCG led to the cross-sectional area (CSA) of *mdx* triceps surae muscle being reduced by 12%, a recovery score of 86% (Tables 1, 2). This suggests that EGCG protects this muscle against dystrophy-related hypertrophy (Sacco et al. 1992). The 40% recovery score of *P*_t_ after oral EGCG administration is close to the 65% that Dorchies et al. (2006) reported previously. However, in their study, oral EGCG treatment did not affect *P*_o_ and the CSA of *mdx* ^*5Cv*^ triceps surae muscle although it improved other parameters, such as the time-to-peak and time for half relaxation from the peak, to normal levels. These differences may be due to the different strains of *mdx* mice that were used (Willmann et al. 2009): Dorchies et al. (2006) used both males and females from the *mdx* ^*5Cv*^ allelic variant, whereas only males from the original *mdx* strain were used in the present study. Recently, Beastrom et al. (2011) found that the *mdx* ^*5Cv*^ strain is more severely affected than the original *mdx* strain. This may be due to the *mdx* ^*5Cv*^ strain being unable to produce either non-muscle dystrophin isoforms or shorter forms of muscle-specific dystrophin that are found in the so-called revertant myofibres of the original *mdx* strain.

The spontaneous locomotor activities of *mdx* mice injected sc with EGCG increased significantly by 36–55% (Fig. 4b; Tables 1, 2). However, mice fed EGCG in their diet showed only a small, non-significant increase (13 ± 8%) in locomotor activity (Fig. 4b; Tables 1, 2). This is in marked contrast with the observation that administration of 0.1% dietary EGCG to age-matched male *mdx* ^*5Cv*^ mice fully normalized their spontaneous activities (Dorchies et al., unpublished data). As discussed above, the inconsistency of these data may be due to variations in the phenotypes of the C57BL/10-*mdx* and C57BL/6-*mdx* ^*5Cv*^ strains with different genetic backgrounds (Beastrom et al. 2011; Fukada et al. 2010; Willmann et al. 2009).

The higher sc EGCG dose was expected to be as effective as the lower dose in improving muscle function, but the substantially increased, near normal, locomotor activities of the treated mice (Table 2) may compete with the recovered stability of the sarcolemma and eventually damage it. This may explain why the 10 mg/kg EGCG dose did not lead to significant or dose-dependent changes in serum CK levels and muscle tensions (Fig. 3a; Table 2). Similar observations have been reported by Call et al. (2008), who found that young *mdx* mice given green tea extract in their diet were much more active than untreated mice in a spontaneous wheel-running assay, but showed no significant reduction in serum CK levels.

We found that oral EGCG administration significantly reduced the number of LF granules in *mdx* diaphragm muscles by 29 ± 3% (Fig. 5 g; Tables 1, 2) and such a reduction is consistent with the 40% increase in the mean antioxidant potential of the plasma of *mdx* ^*5Cv*^ mice given EGCG in their diet (Dorchies et al. 2006). The low dose EGCG injection schedule in the present study did not reduce LF formation significantly in diaphragm muscle, in contrast to our previous report (Nakae et al. 2008) that showed significant reduction by 53 ± 4 and 41 ± 11% in *mdx* diaphragm and soleus muscles, respectively, when the same dose of EGCG was injected for 8 weeks from birth. This suggests that EGCG treatment should be started as early as possible in order to protect diaphragm muscle efficiently from oxidative stress.

Fibrosis is about 25–30% higher in diaphragm and EDL muscles of untreated 8-week-old *mdx* mice compared with that in age-matched normal mice (Fig. 6 g, h; Tables 1, 2). In *mdx* mice, fibrosis was greater in the diaphragm muscle than in the EDL muscle (Fig. 6 g, h; Table 1) because the diaphragm muscle is more susceptible to work-induced injury than other skeletal muscles (Stedman et al. 1991). Orally administered EGCG significantly reduced the amount of connective tissue present in *mdx* EDL muscle by 12 ± 2%, a recovery score of 48% (Fig. 6f, g; Tables 1, 2). In contrast, fibrosis in *mdx* diaphragm muscle was not significantly improved by any of the EGCG schedules we employed, unlike as reported previously (Nakae et al. 2008), where fibrosis was reduced significantly by 41 ± 5 and 20 ± 11% in *mdx* diaphragm and soleus muscles, respectively, when sc injection of EGCG was started from birth.

An interesting observation in treating *mdx* mice with EGCG sc is increased spontaneous locomotor activity (Fig. 4b; Tables 1, 2). We found that the EGCG-mediated alterations of locomotor activity correlated inversely with the numbers of LF granules in diaphragm muscles (Fig. 7b, c) and with fibrosis in EDL muscles (Fig. 7 g). One might expect that the increased locomotor activity would increase the oxidative stress in myofibres, which would in turn lead to the formation of more LF granules (Faist et al. 2001). Because spontaneous locomotor activity is a relatively mild exercise, we hypothesise that the oxidative stress caused by it should be buffered by EGCG, either by acting as a free radical scavenger or indirectly by interacting with high affinity targets (such as superoxide-producing NOX enzyme and signalling molecules in the NF-κB pathway; Khan and Mukhtar 2008; Morré et al*.* 2000; Nishikawa et al. 2007; Shimizu and Weinstein 2005; Steffen et al. 2007, 2008), or both. For EGCG to be effective as a radical scavenger, it must be present at micromolar levels in tissues, whereas its indirect effect can be achieved at much lower tissue concentrations, typically in the range 0.1–1.0 µM (Shimizu and Weinstein 2005). However, the current consensus of opinion is that the in vivo free-radical scavenging activity of flavonoids (which include EGCG) is minor and their interactions with high affinity targets may be more important (Schewe et al. 2008).

The maximum concentration of EGCG in plasma in mice after a single EGCG dose of 10 kg/mg intravenously or 180 mg/kg intragastrically is respectively 2.73 μM (Lambert et al. 2006) or 0.681 μM (calculated from data in Lambert et al. 2006). When EGCG is injected sc, its maximum concentration in plasma is probably lower than 2.73 μM but it is retained in mice longer than EGCG administered intravenously or intragastrically. However, 24–48 h after sc injection, plasma EGCG levels are below the levels of detection. In contrast, in mice given 0.1% EGCG in their diet, the plasma EGCG concentration is about 30 ng/ml (66 nM) in blood samples collected in the light-phase when the mice were consuming much less EGCG than the average daily intake. Consequently this EGCG concentration is probably the minimum level. Nonetheless, the half life of EGCG in plasma is very short (1.4 h; Lambert et al. 2006). The estimated EGCG concentrations (0.1–1.0 μM) in plasma after oral or sc administration are sufficient for it to interact with high affinity targets such as 67LR (*K*_d_ = 39.9 nM; Tachibana et al. 2004) and NOX enzyme (IC_50_ = 3.5 μM; determined for EGCG as an inhibitor by Steffen et al. 2008), both of which are over-expressed in dystrophic muscle cells and tissues (Dorchies et al. 2009; Shkryl et al. 2009; Whitehead et al. 2010). The variations in the amounts of the high affinity targets in different types of muscles may also affect the efficacy of EGCG.

The inverse correlation of the EGCG-mediated alterations of locomotor activity with the numbers of LF granules in diaphragm muscles (Fig. 7b, c) implies that mice with higher locomotor activities form fewer LF granules in their muscles. This relationship is independent of routes and doses of EGCG administration. The diaphragm, which contracts continuously, may resist the oxidative stress caused by locomotion more than locomotor muscles, such as EDL muscle. The correlation for the EDL is dependent on the routes and doses of EGCG administration: the 0.1% oral EGCG treatment led to a similar correlation as in the diaphragm, but 10 mg/kg EGCG injected sc resulted in an opposite correlation (Fig. 7e, f). This suggests that EDL muscles in *mdx* mice given 10 mg/kg EGCG sc, whose locomotor activities are close to normal levels (Fig. 4b; Tables 1, 2), require high amounts of EGCG to counteract the effects of activity-mediated oxidative stress. Nonetheless, the formation of slightly more LF granules in EDL muscles in *mdx* mice injected sc with 10 mg/kg EGCG is probably attributable to such stress (Fig. 5 h; Tables 1, 2). The different resistance of EDL and diaphragm muscles to oxidative stress may be due to different expression of high affinity targets for EGCG.

In summary, sc injection of 5 mg/kg EGCG 4 times a week led, compared to the other two dose–route protocols, to the highest average reduction in serum CK levels, the second best improvement in locomotor activity and *P*_i_ and *P*_o_, but no significant reduction in the amount of LF in diaphragm muscle. Doubling the sc dose of EGCG resulted in the greatest increase in locomotor activity. However, this improvement was accompanied by an insignificant reduction in diaphragm LF, an insignificant increase in EDL LF and the lowest increases in *P*_i_ and *P*_o_. Oral administration of EGCG in the diet produced the greatest reduction in the amount of LF formed in diaphragm muscle, the greatest improvement in muscle tensions and hypertrophy, a significant reduction in the fibrosis of the EDL muscle and serum CK levels, but a relatively poor improvement in locomotor activity. The greater efficacy of the oral route is probably due to maintaining a plasma concentration of EGCG of at least 66 nM throughout the treatment period.

We conclude that the efficacy of EGCG found in this and previous studies for improving the structure and function of muscles in *mdx* mice suggests that EGCG may be of benefit for DMD patients.
